# Comparative Analysis of Alpha-1 Orthosteric-Site Binding by a Clade of Central American Pit Vipers (Genera *Atropoides, Cerrophidion, Metlapilcoatlus*, and *Porthidium*)

**DOI:** 10.3390/toxins15080487

**Published:** 2023-08-02

**Authors:** Lee Jones, Callum Waite, Edgar Neri-Castro, Bryan G. Fry

**Affiliations:** 1 Venom Evolution Laboratory, School of the Environment, University of Queensland, St Lucia, Queensland 4072, Australia; callum.waite@csiro.au; 2 Facultad de Ciencias Biológicas, Universidad Juárez del Estado de Durango, Av. Universidad s/n. Fracc. Filadelfia, Gómez Palacio 35010, Dgo., Mexico; nericastroedgare@gmail.com; 3 Instituto de Biotecnología, Universidad Nacional Autónoma de México, Avenida Universidad 2001, Chamilpa, Cuernavaca 62210, Mor., Mexico

**Keywords:** venom, neurotoxic, nicotinic acetylcholine receptor

## Abstract

The distribution and relative potency of post-synaptic neurotoxic activity within Crotalinae venoms has been the subject of less investigation in comparison with Elapidae snake venoms. No previous studies have investigated post-synaptic neurotoxic activity within the *Atropoides*, *Metlapilcoatlus*, *Cerrophidion*, and *Porthidium* clade. Given the specificity of neurotoxins to relevant prey types, we aimed to uncover any activity present within this clade of snakes that may have been overlooked due to lower potency upon humans and thus not appearing as a clinical feature. Using biolayer interferometry, we assessed the relative binding of crude venoms to amphibian, lizard, bird, rodent and human α-1 nAChR orthosteric sites. We report potent alpha-1 orthosteric site binding in venoms from *Atropoides picadoi*, *Metlapilcoatlus occiduus*, *M. olmec*, *M. mexicanus*, *M. nummifer*. Lower levels of binding, but still notable, were evident for *Cerrophidion godmani*, *C. tzotzilorum* and *C. wilsoni* venoms. No activity was observed for *Porthidium* venoms, which is consistent with significant alpha-1 orthosteric site neurotoxicity being a trait that was amplified in the last common ancestor of *Atropoides/Cerrophidion/Metlapilcoatlus* subsequent to the split by *Porthidium*. We also observed potent taxon-selective activity, with strong selection for non-mammalian targets (amphibian, lizard, and bird). As these are poorly studied snakes, much of what is known about them is from clinical reports. The lack of affinity towards mammalian targets may explain the knowledge gap in neurotoxic activity within these species, since symptoms would not appear in bite reports. This study reports novel venom activity, which was previously unreported, indicating toxins that bind to post-synaptic receptors may be more widespread in pit vipers than previously considered. While these effects appear to not be clinically significant due to lineage-specific effects, they are of significant evolutionary novelty and of biodiscovery interest. This work sets the stage for future research directions, such as the use of *in vitro* and *in vivo* models to determine whether the alpha-1 orthosteric site binding observed within this study confers neurotoxic venom activity.

## 1. Introduction

Snake venom toxins have evolved to disrupt a range of pathophysiological targets to immobilise prey [1]. One way in which snake venoms can incapacitate prey is targeting the nervous system, causing flaccid or spastic paralysis. While post-synaptic neurotoxicity is typically found within Elapidae and Colubridae, there is growing evidence for widespread post-synaptic neurotoxic activity within viperid snakes [2,3,4,5].

Snake venom neurotoxins can cause flaccid paralysis via the inhibition of the orthosteric site of post-synaptic muscle-type α-1 nicotinic acetylcholine receptors (nAChRs). Snake toxins targeting the α-1 nAChR have evolved independently on three separate occasions: 3FTx (three-finger toxins) which are abundant in many elapid and colubrid snake venoms [6,7,8,9]; phospholipase A_2_ (PLA_2_) toxins such as those from *Bitis* [10,11,12]; *de novo* evolution of neurotoxic peptides within the propeptide region of the gene encoding for natriuretic peptides, which are present in linear forms, including azemiopsins from *Azemiops* [10] and waglerins from *Tropidolaemus* venoms [12,13]. While these toxins are responsible for lethal effects in some venoms, as seen in elapid snakes, they may be highly specific for non-mammalian lineages [4,7,8,14]. Consequently, as many snake venom effects are known only from clinical reports, the non-mammalian selective neurotoxic activity may not be noted in case studies. Such selectivity may be present within a higher order lineage; this can be seen in the venom of the snake-specialist *Ophiophagus hannah*, which is not only selective for reptiles, but possesses even higher selectivity towards snake nAChRs relative to lizard [15]. 

Coagulotoxicity dominates the clinical landscape for viper venoms, and consequently this aspect of venom biochemistry has been the focus of extensive research [16,17,18,19,20,21,22,23,24,25,26,27,28,29,30]. Of the neurotoxins, the effect of presynaptic neurotoxins is significant in human envenomations [31,32]; as such, the focus of viper venom neurotoxicity research has been on presynaptic toxins. Notably, crotoxin isolated from *Crotalus dissurus terrificus*, and subsequent isoforms found in other species, cause flaccid paralysis by preventing the release of acetylcholine [33,34,35,36,37,38,39,40,41]. While post-synaptic neurotoxicity has long been known from *Tropidolaemus* [5] and *Azemiops* [10], it has only been described recently from few pit vipers such as *Bothriechis*, *Calloselasma*, and *Tropidolaemus* [3,5,36]. 

Venom from the *Atropoides*, *Cerrophidion*, *Metlapilcoatlus*, *Porthidium* clade have been subject to limited research, with mainly proteomics and few coagulotoxic investigations performed [28,42,43,44,45,46,47,48]. Proteomic analysis shows that venom composition remains similar between species, with metalloproteinases (SVMP), serine proteases (SVSP) and PLA_2_s comprising the majority of toxins present. Remarkably, Angulo’s analysis of *M. mexicanus* venom revealed the presence of a 3FTx in the venom proteome [47]. However, the extent of the functional biological activity of this toxin within *M. mexicanus’s* venom remains unknown. 

Functional coagulotoxic research has revealed that, despite proteomic similarity, venoms are active on a plethora of pathophysiological targets within the coagulation cascade. For instance, *Porthidium volcanicum* was uniquely procoagulant within the clade, activating FXII to form strong blood clots *in vitro* [28]. While no neurotoxic symptoms have been reported clinically, humans are of course not a natural prey item to these species, and many of the species take a large proportion of non-mammalian prey in their diet. Given that diet is a main driver of venom evolution, as well as the specificity of α-neurotoxins and the diversity of venom effects seen within this clade, the absence of symptoms in bite victims does not exclude the presence of neurotoxins [49]. Therefore, the functional testing of neurotoxicity against relevant prey types is necessary to understand not only the evolutionary history of a venom, but also the ecological role it plays. 

To understand the potential unique neurotoxic effects of venoms, as well as species-specific activity, we used biolayer interferometry to measure binding to the alpha-1 orthosteric site of a variety of relevant prey mimotopes. Venoms from these genera have never been assessed for neurotoxicity, providing an opportunity to describe new venom phenotypes. 

## 2. Results

**Figure 1:**
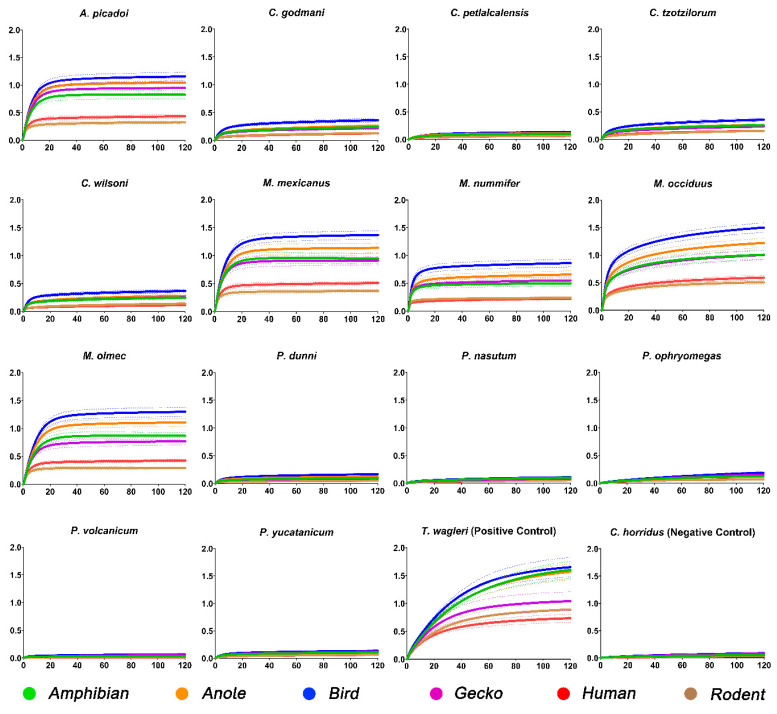
Comparison of wavelength (nm) curves of *Atropoides*/*Cerrophidion*/*Metlapilcoatlus*/*Porthidium* venom over a 120 s period. To keep controls consistent to Crotalinae, *T. wagleri* was used as the positive control and *C. horridus* as the negative. All venoms were tested against amphibian (green), anole (orange), bird (blue), gecko (purple), human (red), and rodent (brown) mimotopes. Dots surrounding the mean lines represent the standard error of the mean (SEM). Each venom was tested in triplicate (*n* = 3).

Of the 14 species tested, we observed alpha-1 orthosteric site binding by eight species (Figure 1). All *Metlapilcoatlus* species included within this study showed potent binding similar to the positive control, *T. wagleri*. *Metlapilcoatlus occiduus* was the most potent, displaying strong binding across all taxon receptors, but with non-mammalian targets bound much stronger than rodent or human. Within *Cerrophidion*, lower levels of binding were seen for *C. godmani*, *C. wilsoni*, and *C. tzotzilorum*. Whereas *C. petlalcalensis* showed no significant binding. None of the *Porthidium* species tested presented significant binding to any mimotope tested. 

**Figure 2:**
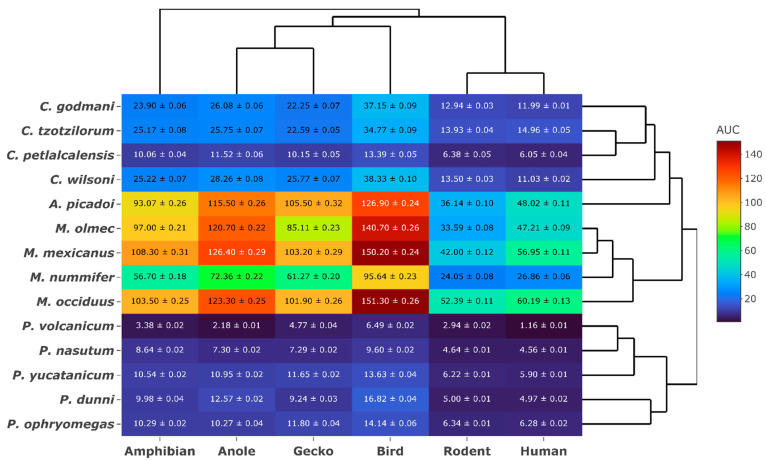
Heat map comparison of *Atropoides*/*Cerrophidion*/*Metlapilcoatlus*/*Porthidium* species. Values are AUC ± SEM and are derived from binding-rate curves; warmer colours indicate stronger binding whereas cooler colours indicate weaker/no binding. Snake phylogeny is based on Alencar et al. [50]. Species phylogeny was obtained by entering the taxa into www.timetree.org (accessed 1 May 2023).

AUC (Area under curve) values indicate taxon-specific activity where binding was observed in relation to snake phylogeny (Figure 2). The highest relative binding was observed with the bird mimotope, while lower binding is reported for mammalian mimotopes. *Metlapilcoatlus mexicanus* showed the greatest increase in binding (318%) from the rodent to the bird receptors. 

## 3. Discussion

This study reports that venoms from all *Metlapilcoatlus* species tested show high affinity for alpha-1 orthosteric sites, with *M. occiduus*, *M. olmec*, and *M. mexicanus* being equipotent to the results obtained for the Asian pit viper *Tropidolaemus wagleri* (Figure 1), which is well-documented as being neurotoxic [5,12,14]. In addition to the overall levels of binding, *Metlapilcoatlus* and *Atropoides* species also had a faster onset in comparison to that of *T. wagleri*. This is consistent with *Atropoides* and *Metlapilcoatlus* being sit-and-wait ambush-predators. As they are morphologically stout, and consequently slow-moving (but fast-striking), they have limited abilities to pursue prey. Therefore, prey escape is a strong selection pressure acting upon these venoms, which is consistent with our results of fast-acting neurotoxins. This is congruent with other species of venomous animal, in which venoms are selected for very fast-onsetting neurotoxins due to the potential for prey escape (such as cone snails) [51,52] or prey retaliation (seen in the blue long-glanded coral snake *Calliophis bivirgatus* which feeds on other venomous snakes) [53]. 

Consistent with the hypothesis that prey escape potential is a strong selection pressure for these venoms, all *Atropoides* and *Metlapilcoatlus* venoms displayed the strongest affinity toward the bird receptor (Figure 1 and Figure 2). Based on few limited diet studies, *Metlapilcoatlus* are thought to be generalists that opportunistically feed upon diverse prey, with amphibians, lizards, small passerines, orthopterans, and mammals recovered from dietary studies [54]. Venom from these species reflects *Calloselasma rhodostoma*, an Asian pit viper species with similar morphology, and inhabits the same niche as *Atropoides*, *Metlapilcoatlus* and *Cerrophidion*, who have also been shown to possess post-synaptic neurotoxic activity [55]. This research further emphasises the link between diet and venom activity, highlighted by the specificity of α- neurotoxins to relevant prey receptors. In particular, the greatest increase in binding was seen between the rodent and the bird receptors, indicating these toxins are specific for non-mammalian targets (Figure 2). Furthermore, the low binding observed in the human receptor may explain the absence of neurotoxic symptoms described within clinical envenomation reports [56,57]. As humans are not prey items for these species, there is low selection pressure for neurotoxins to act upon human α-1 binding regions.

The toxin types responsible for the alpha-1 orthosteric site binding observed in this study are unknown, just as they are for Central American pit vipers within the *Bothriechis* genus [3] or the Asian pit viper *Calloselasma rhodostoma* [5]. While 3FTx has been shown in very small quantities within the venom proteome of *M. mexicanus*, the levels present are far too low to explain the level of activity observed in this study. As post-synaptic PLA_2_ have previously been isolated from *Bitis* venoms [58] and post-synaptic neurotoxicity is broadly present within that genus [2], as well as other viperine genera [4], it is hypothesised that similar PLA_2_ are responsible for the alpha-1 orthosteric site binding activity in pit vipers such as the species in this study. This may also be the case for the aforementioned activity in other pit vipers, such as the American *Bothriechis* [3] and the Asian *Calloselasma* [5].

In contrast to the fast and potent activity of *Atropoides* and *Metlapilcoatlus*, only comparatively low binding was observed for *Cerrophidion* species, with *C. petlalcalensis* devoid of any discernible significant activity. Similarly, all *Porthidium* species lacked notable alpha-1 orthosteric site binding. This pattern is congruent with that shown for relative anticoagulant actions through the inhibition of clotting factors or destruction of fibrinogen. The clades in this study that were the least neurotoxic, were shown to be the most potently anticoagulant through enzyme-inhibition or fibrinogen-destruction [28]. Reciprocally, the species shown to be the most potently neurotoxic in this study, were those in the clade previously shown to be anticoagulant through the pseudo-procoagulant mechanism whereby fibrinogen is converted into abnormal, weakly structured fibrin clots that break down much more rapidly than endogenously produced fibrin clots, and therefore ultimately contribute to a net anticoagulant state by depleting fibrinogen levels [28]. 

Phylogenetic reconstruction of the evolutionary history and identification of the toxins responsible for the alpha-1 orthosteric site binding activity is necessary to ascertain if these toxins are homologous to those responsible for post-synaptic neurotoxicity in other vipers (e.g., azemiopsin/waglerin peptides and PLA_2_s). Regardless, as both *Atropoides* and *Metlapilcoatlus* show significant neurotoxicity, but are not sister genera, this suggests that post-synaptic neurotoxicity was present in the last common ancestor of this clade, and was independently amplified as a trait in *Atropoides* and *Metlapilcoatlus*. In light of this, it is hypothesised that the toxins responsible will be shared with *Bothriechis*, the other genus from the Americas shown to have significant levels of post-synaptic neurotoxicity [3]. Future studies should be conducted to provide a more definitive answer of neurotoxic activity, such as utilizing both *in vitro* and *in vivo* models to determine if the binding observed within this assay confers post-synaptic neurotoxicity. 

## 4. Conclusions

We report widespread alpha-1 orthosteric site binding activity for *Atropoides* and *Metlapilcoatlus* and lower levels in *Cerrophidion* species. In addition, we have shown the neurotoxic toxins present are more selective towards non-mammalian prey. Further ecological and natural history studies are required to link the functional testing of venom to life history in order to understand the evolutionary history of these genera. The comparatively low levels on human receptors are congruent with these effects not being usual clinical features.

## 5. Materials and Methods

### 5.1. Venoms 

All venom work was performed under the University of Queensland Animal Ethics Approval 2021/AE000075 and UQ Biosafety Committee Approval # IBC/134B/SBS/2015.

Venoms were sources from fourteen captive snakes used within this study: *Atropoides picadoi* (Costa Rica), *Cerrophidion godmani* (Mexico), *Cerrophidion petlalcalensis* (San Andres Tenejapan, Veracrus, Mexico), *Cerrophidion tzotzilorum* (San Cristobal, Chiapas, Mexico), *Cerrophidion wilsoni* (Honduras), *Metlapilcoatlus mexicanus* (Chiapas, Mexico), *Metlapilcoatlus nummifer* (Veracruz, Mexico), *Metlapilcoatlus occiduus* (Mapastepec, Chiapas, Mexico), *Metlapilcoatlus olmec* (Los Tuxtlas, Veracruz, Mexico), *Porthidium dunni* (Oaxaca, Mexico), *Porthidium nasutum* (Mexico), *Porthidium ophryomegas* (Costa Rica), *Porthidium volcanicum* (Costa Rica), and *Porthidium yucatanicum* (Solidaridad, Quintana Roo, Mexico). Lyophilized venoms were reconstituted in double-deionised water (DDH2O) and centrifuged (4 °C, 10 min, 14,000 RCF). Working stock solutions were made to a concentration of 1 mg/mL 50% DDH2O and 50% glycerol. Concentrations of the working stock were determined in triplicate using a NanoDrop 2000 (Thermofisher, Sydney, Australia) at 280 nm wavelength. 

### 5.2. Mimotope Preparation

Following previously validated methods [15], a thirteen–fourteen amino acid chain mimotope of the vertebrate α-1 nAChR orthosteric site was designed using publicly available sequences of cholinergic receptors from amphibian, gecko, anole, bird, rodent, and human receptors. Receptor sequences were sourced from Genbank and UniProt, and developed by GeneticBio (Shanghai, China). Mimotope dry stocks were reconstituted in 100% dimethyl sulfoxide (DMSO) and diluted 1:10 to produce a 50 µg/mL working stock in 10% DMSO.

### 5.3. Biolayer Interferometry

In order to test the neurotoxic activity, biolayer interferometry (BLI) was used on the Octet HTX system (ForteBio, Fremont, CA, USA). BLI is an optical technique that measures the thickness of biomolecules accumulated on an optical-fibre-coated biosensor. Bound molecules cause a spectral shift in the wavelength of light being reflected through the fibre-optic biosensor, allowing a quantified measure of binding. Full details of the assay used within this study can be found in the validated protocol [15]. Prior to experimentation, Streptavidin biosensors were hydrated in a running buffer (Dulbecco’s phosphate-buffered saline (DPBS) with 0.1% BSA and 0.05% Tween-20) for 30–60 min while shaken at 2.0 revolutions per minute (RPM). Venom (analyte) samples were diluted to make an experimental concentration of 50 µg/mL per well and mimotope aliquots were diluted to a final concentration of 1 µg/mL per well. A standard acidic glycine buffer solution (10 mM glycine (pH 1.5–1.7) in DDH2O) was used to cause the dissociation of analytes.

### 5.4. Data Analysis

Data processing was performed in accordance with the validated assay. Data were extracted directly from the Octet HTX and imported into Prism 9.5.0 software (GraphPad Software Inc., La Jolla, CA, USA) for data analysis. This study utilized area under the curve (AUC) for ease of data viewing in a heatmap format. All raw data are available in supplementary material.

## Data Availability

All raw data are available in the Supplementary Materials.

## Supplementary Materials

The following supporting information can be downloaded at https://www.mdpi.com/article/10.3390/toxins15080487/s1. Data can be viewed within Supplementary File S1.
